# Classic Kaposi's sarcoma in Italy, 1985–1998

**DOI:** 10.1038/sj.bjc.6602265

**Published:** 2004-11-30

**Authors:** L Dal Maso, J Polesel, V Ascoli, P Zambon, M Budroni, S Ferretti, R Tumino, G Tagliabue, S Patriarca, M Federico, M Vercelli, A Giacomin, G Vicario, F Bellù, F Falcini, E Crocetti, V De Lisi, S Vitarelli, S Piffer, F Stracci, D Serraino, G Rezza, S Franceschi

**Affiliations:** 1 Servizio di Epidemiologia e Biostatistica, Centro di Riferimento Oncologico IRCCS, Via Pedemontana Occ.le 12, 33081 Aviano, Italy; 2 Dipartimento di Medicina Sperimentale e Patologia, Università La Sapienza, Viale Regina Elena 324, 00161 Rome, Italy; 3 Università di Padova, Registro Tumori del Veneto, Via Gattamelata 64, 35128 Padua, Italy; 4 Registro Tumori della Provincia di Sassari, Centro Multizonale di Osservazione Epidemiologica, Via Tempio 5, 07100 Sassari, Italy; 5 Registro Tumori della Provincia di Ferrara, Sezione di Anatomia, Istologia e Citologia Patologica, Via Fossato di Mortara 64, 44100 Ferrara, Italy; 6 Registro Tumori Azienda Ospedaliera Civile ‘MP Arezzo’, Via Dante 109, 97100 Ragusa, Italy; 7 Registro Tumori Lombardia – Provincia di Varese, Istituto Nazionale Tumori, Via Venezian 8, 20133 Milan, Italy; 8 Registro Tumori del Piemonte, Ospedale S Giovanni Antica Sede, Via S Francesco da Paola 31, 10123 Turin, Italy; 9 Registro Tumori della Provincia di Modena, Policlinico, Via Dal Pozzo 71, 41100 Modena, Italy; 10 Registro Tumori Ligure, Istituto Nazionale per la Ricerca sul Cancro, Largo Rosanna Benzi 10, 16132 Genoa, Italy; 11 Registro Tumori della Provincia di Biella, Azienda Sanitaria Locale 12, Via Don Sturzo 20, 13900 Biella, Italy; 12 Registro Tumori del Friuli-Venezia Giulia, Agenzia Regionale di Sanità, Piazzale S Maria della Misericordia 15, 33100 Udine, Italy; 13 Registro Tumori dell'Alto Adige, Corso Italia 13/M, 39100 Bolzano, Italy; 14 Registro Tumori della Romagna, Istituto Oncologico Romagnolo, Dipartimento Oncologico, Ospedale Pierantoni, Via Forlanini 11, 47100 Forlì, Italy; 15 Epidemiologia clinica e descrittiva, Centro Studio e Prevenzione Oncologica, Via di S Salvi 12, 50135 Florence, Italy; 16 Registro Tumori della Provincia di Parma, Ospedale di Parma, Via Gramsci 14, 43100 Parma, Italy; 17 Registro Tumori di Macerata, Università di Camerino, Viale E Betti 3, 62032 Camerino, Italy; 18 Registro Tumori della Provincia di Trento, Osservatorio Epidemiologico, Via Gilli 3, 38100 Trento, Italy; 19 Registro Tumori Umbro di Popolazione, Università di Perugia, Via del Giochetto, 06123 Perugia, Italy; 20 Dipartimento di Epidemiologia, INMI ‘L Spallanzani’ IRCCS, Via Portuense 292, 00149 Rome, Italy; 21 Istituto Superiore di Sanità, Viale Regina Elena 299, 00161 Rome, Italy; 22 International Agency for Research on Cancer, 150 cours A Thomas, 69372 Lyon, France

**Keywords:** classic Kaposi's sarcoma, incidence, Cancer Registries, Italy

## Abstract

To evaluate incidence rates (IRs) of classic Kaposi's sarcoma (CKS) in Italy after the spread of AIDS, we distinguished CKS from AIDS-related KS (AKS) using an ‘*ad hoc*’ record linkage procedure between 15 Cancer Registries (CRs) (21% of the Italian population) and the national AIDS Registry. Between 1985 and 1998, 874 cases of CKS and 634 cases of AKS were diagnosed in the study areas. CKS accounted for 16 and 27% of KS cases below 55 years of age in men and women, respectively, but for 91 and 100% of those above age 55. The IRs for CKS were 1.0/ in men and 0.4/100 000 in women, but they varied between 0.3 in Umbria and 4.7 in Sassari in men, and between 0.1 in Parma and 1.7 in Sassari in women. IRs of CKS in both genders were stable between 1985–1987 and 1993–1998. In Northern and Central CRs the IR (adjusted for age and gender) for CKS was 0.5 in individuals born in the same area, but 1.6 in individuals born in Southern Italy or in the Islands (rate ratio=3.2) suggesting that KS-associated herpesvirus, the cause of KS, is acquired early in life.

Before the AIDS epidemic, Kaposi's sarcoma (KS) occurred sporadically in Europe, most notably in elderly men of Mediterranean or Middle Eastern origin (classic KS, CKS) (Franceschi and Geddes, 1995). Some increase in incidence was reported in the 1960s and 1970s and attributed to the increase in immunosuppressive treatment (e.g. corticosteroid use) (Dictor and Attewell, 1988).

Since the 1980s, consequent on the AIDS epidemic, KS incidence has increased in all Western countries (Dal Maso *et al*, 1999), mainly due to the high frequency of AIDS-associated KS (AKS) among HIV-infected homosexual and bisexual males (IARC, 1996). KS-associated herpesvirus (KSHV or human herpesvirus type 8, HHV8) is recognised as the necessary, though not sufficient, cause of all clinical variants of KS (Moore and Chang, 1998; Iscovich *et al*, 2000).

Owing to the rise in AKS incidence, the evaluation of CKS incidence trends has become difficult. KS rates in older persons (e.g. ⩾65 years), who have an extremely low prevalence of HIV/AIDS, have been used as a proxy for CKS rates (Geddes *et al*, 1994; Hjalgrim *et al*, 1998). Other studies manually reviewed medical records of KS cases to distinguish major KS types (Ascoli *et al*, 2001; Santarelli *et al*, 2001). Automatic linkage procedures (Franceschi *et al*, 2003) provide a powerful tool to distinguish CKS from AKS in populations where Cancer Registries (CRs) and HIV/AIDS Registries both exist (Guttman-Yassky *et al*, 2003), and we applied this approach in our Italian study.

## MATERIALS AND METHODS

CKS and AKS were distinguished using the record-linkage procedure of Cancer and AIDS Registries Linkage (CARL) study (Dal Maso *et al*, 2003). Several independent CRs, covering a population of 11.6 million (21% of the total Italian population), have produced accurate KS incidence rates (IRs) for the mid-1990s (Parkin *et al*, 2002; Zanetti *et al*, 2002). They covered the ‘North-East network’, including the Friuli-Venezia Giulia region and the provinces of Trento and Alto Adige, the regions of Romagna and Umbria, part of the Veneto region, the municipality of Turin, the provinces of Biella, Genoa, Varese, Parma, Modena, Ferrara, Macerata, Florence and Prato, Sassari and Ragusa. CRs varied in size, ranging from populations of approximately 190 000 to 2 million, as well as in the number of registration years available (Parkin *et al*, 2002; Dal Maso *et al*, 2003).

AIDS notification in Italy started in 1982 and has been mandatory since 1986. By the end of the year 2000, 47 503 AIDS cases had been recorded according to the clinical standard definitions (Istituto Superiore di Sanità, 2003).

An ‘*ad hoc*’ software application (Software for Automated Linkage in Italy, SALI) was developed to perform the record linkage procedure (Dal Maso *et al*, 2001a). Briefly, records from the Italian AIDS registry (RAIDS) and CRs are linked by first and last name, and by date of birth. Satisfaction of the name-date algorithm requires: (a) that the records are identical for at least one critical field; and (b) that the other two critical fields, if not identical, differ only in prescribed ways. Personal identifiers are removed during linkage procedures; thus, the staff of each registry is blinded to which persons have been linked. SALI has been shown to have a very high sensitivity and specificity in the Italian context, and it is available upon request (Dal Maso *et al*, 2001a).

The present study was restricted to people who: (1) were diagnosed with KS between 1985 and 1998; (2) reported legal residence in areas covered by CRs; and (3) had KS in periods deemed complete by both CRs and RAIDS. All ages were included.

KS cases were considered AIDS-related when: (a) they occurred in patients with AIDS notified at RAIDS; (b) AIDS was one of the three causes mentioned on the death certificate; (c) HIV-infection was mentioned in any additional medical record available at the CR (e.g. HIV-positive test or mention of AIDS-related disease at hospital admission or death). A total of 16 unlinked KS were reclassified as AKS according to criteria (b) and (c), otherwise the KS cases were considered as CKS.

Nine KS cases were excluded because they arose in the transplant patients, information recorded in only three CRs (Genoa, Sassari and Veneto). One KS case notified only by death certificate and three for whom the type of diagnosis (e.g. clinical, histological, etc) was unknown were also excluded.

Standardised IRs were computed by gender, place of residence, place of birth and overall using the direct method (Breslow and Day, 1987) and the 1991 Italian population as standard population. Rate ratios (RR) adjusted by age and gender were calculated by place of birth, and 95% percent confidence intervals (CI) were computed according to the Poisson distribution (Breslow and Day, 1987). To evaluate temporal trends, IRs were computed separately into three periods: (a) 1985–1987 (Parkin *et al*, 1992); (b) 1988–1992 (Parkin *et al*, 1997) and (c) 1993–1998 (Parkin *et al*, 2002).

## RESULTS

Between 1985 and 1998, 874 CKS and 634 AKS were reported to Italian CRs (Table 1). The age distribution of CKS was similar for men and women, the median age was 72 years (range 18–95), and only 119 (14%) occurred in persons below age 55. Conversely, AKS occurred at a median age of 36 (range 19–83) and only 49 (8%) were diagnosed at age 55 or above.

With respect to site of cancer presentation, skin predominated in both CKS (88%) and AKS (86%) but skin of the leg and hip was more frequently involved in CKS than in AKS (Table 1). In total, 10% of AKS was diagnosed in the lip, oral cavity, pharynx and digestive organs compared to only 3% of CKS.

**Figure 1:**
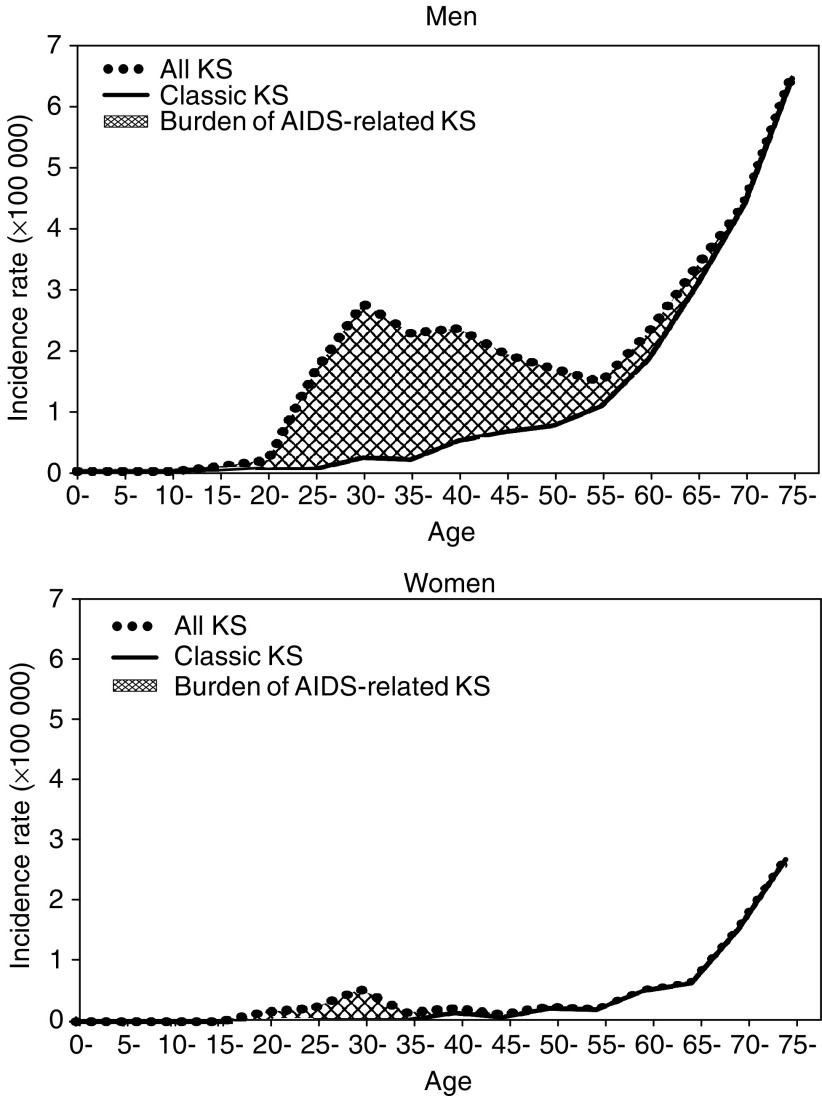
Incidence rates of classic Kaposi's sarcoma (KS) and all KS by age in 15 Italian Cancer Registries and burden of AIDS-related KS in men and women, 1985–1998.

Figure 1 shows the age-specific IRs of CKS and all KS in all Italian study areas in the period 1985–1998. It is noteworthy that in Italy CKS accounted for 91.0 and 100% of new cases of KS, respectively, in men and women aged 55 years or older in Italy, but only 15.7 and 26.9%, respectively, below that age. No significant correlation emerged between IRs of CKS and AKS in different areas (Spearman coefficient, *r*=−0.16 in men and 0.38 in women, data not shown).

Table 2 shows CKS IRs by gender and place of residence. Overall, the IRs were 0.98/100 000 men and 0.41/100 000 women. However, a wide heterogeneity emerged, with IRs between 0.3 in Umbria (Central Italy) to 4.7 in Sassari (Sardinia, Southern Italy) in men, and between 0.1 in Parma (Northern Italy) to 1.7 in Sassari in women (Table 2). Central Italy showed the lowest IRs while, in Northern Italy, the IRs in Ferrara were similar to those in Ragusa (Sicily) and significantly higher than those in nearby areas in both genders. Three-fold higher IRs were found in the two CRs from Southern Italy, mainly on account of particularly elevated IRs in Sassari.

A high positive correlation (*r*=0.66) between IRs in men and women emerged. Thus, male-female ratios were generally between 2.0 and 2.5 in most of the areas, despite some probability of random variation.

The association of place of birth and CKS was evaluated in Northern and Central CRs (Table 3). Among CKS cases in Northern and Central Italy, the IR was 0.5 for individuals born in the same area, while it was 1.6 for individuals born in Southern Italy or in the Islands (RR=3.2; 95% CI: 3.2–3.3). The RRs for individuals born in the South or in the Islands were particularly elevated in the North-East (7.0) and Umbria (9.5), but close to unit in Ferrara (1.7) and Varese (1.6).

**Figure 2:**
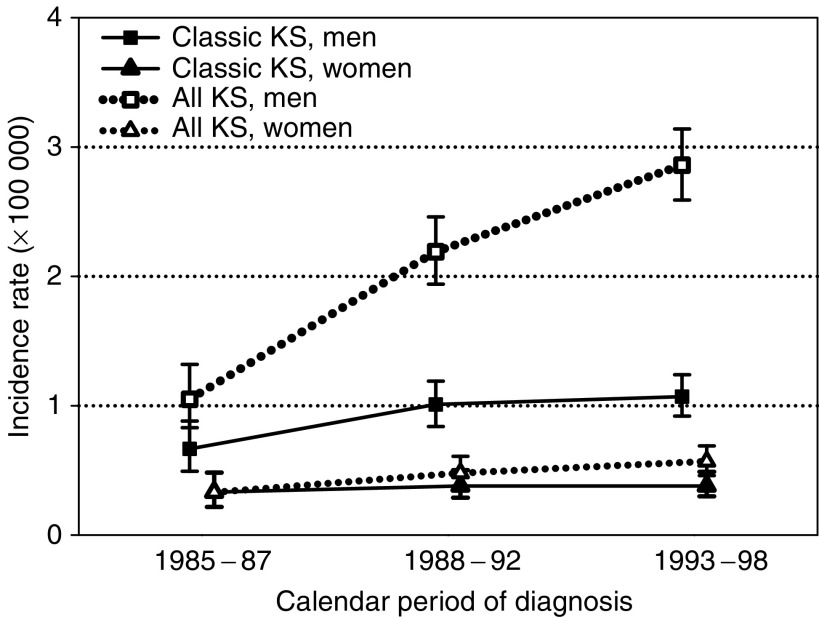
Age-standardised incidence rates (according to Italian population, 1991) and 95% confidence intervals of classic Kaposi's sarcoma (KS) and all KS by year at diagnosis in seven Italian Cancer Registries (analyses have been restricted to Cancer Registries active since 1985 (i.e., Turin, Genoa, Varese, Parma, Romagna, Florence, and Ragusa), 1985–1998.

Figure 2 shows trends in the CKS incidence and, for comparison purposes, for all KS in the seven CRs that had been active since 1985. CKS IRs in men slightly increased between 1985–1987 (IR=0.7) and 1988–1992 (1.0) but did not change thereafter (1.1 in 1993–1998). In women, the IRs were between 0.3 and 0.4 throughout the considered period. By contrast, consequent to the steady increase of people with HIV/AIDS, the incidence of all KS increased from 1.1 and 0.3 in 1985–1987 to 2.9 and 0.6 in 1993–1998 in men and women, respectively.

## DISCUSSION

Our study is one of the first attempts (Guttman-Yassky *et al*, 2003) to describe the epidemiology of CKS in a large population after the huge increase of KS caused by the AIDS epidemic (Dal Maso *et al*, 2001b). A many-fold higher incidence of CKS, compared to Northern Europe, the United States and Australia had already been recorded in Italy before 1985 (Geddes *et al*, 1994; Franceschi and Geddes, 1995; Cottoni *et al*, 1996).

A relatively high incidence of CKS was confirmed in the present study for the 1985–1998 period and the evidence of a broad within-country variation in both genders was expanded. The new CR in Sardinia, one of the only two available in the South and Islands, confirmed previous reports (Santarelli *et al*, 2001; Atzori *et al*, 2004) and showed a four-fold excess, compared to North (Ascoli *et al*, 2003) and Central Italy, where the majority of CRs are concentrated. However, high rates of CKS are also recorded in some areas in the North, notably in Ferrara, in the delta of the Po (Parkin *et al*, 2002).

The reasons for such variations in CKS are not well understood. In our present study, we showed that the incidence of CKS was mainly determined by place of birth but not by the place of living. Being born in the South of Italy was associated with a three-fold increased risk of CKS among individuals who lived in the North and Centre. The massive waves of migration from the rural South to industrial towns in the North, such as Genoa, Turin and Varese during the last century probably account for the moderate excess of CKS in the corresponding CRs compared to the national average.

Comparison with other countries is hampered by the difficulty in distinguishing CKS and AKS. IRs for all KS, in the second half of the 1990s, are available from 186 registries for 57 countries (Parkin *et al*, 2002). These range, in men, from less than 0.5/100 000 in most CRs in Asia and Northern Europe, relatively spared by the AIDS epidemic, to 16.7/100 000 in non-Hispanic Whites in San Francisco, California and 50.8/100 000 in Harare, Zimbabwe (Parkin *et al*, 2002). Apart from Italy, recent population-based CKS IRs are only available for Israeli Jews. After excluding AKS cases, Guttman-Yassky *et al* (2003) reported for 1960–1998 a CKS incidence of 2.1/100 000 for men and 0.8/100 000 for women, standardised using the world standard population. Using the same standard population, the IRs for Italy (0.6 in men and 0.2 in women) was lower than in Israel, but in Sardinia (2.6 in men and 0.6 in women) was similar to Israel. Substantial variations in the incidence of CKS were shown in Israel by country of origin, with approximately two-fold higher IRs among Jews from Iraq, Morocco, and Romania compared to Jews who were born in Israel (Guttman-Yassky *et al*, 2003).

Our findings from Italy suggest substantial stability of CKS incidence between 1985 and 1998, as in Israel in the same period, when various Israeli subpopulations were taken into consideration (Guttman-Yassky *et al*, 2003). A male-to-female ratio of 2-to-3 is also similar in Italy and Israel.

Despite some limitation of available data, the seroprevalence of KSHV antibodies in various geographic areas is correlated with the frequency of KS not associated to AIDS. The seroprevalence of KSHV antibodies is more than 25% in Africa and below 10% in the United States and Europe (Whitby and Boshoff, 1998). Substantial variations, consistent with those reported here for CKS incidence, were also found between Italian regions, with seroprevalence below 10% in certain areas of the North (Calabrò *et al*, 1998; Whitby *et al*, 1998) but higher than 30% in the Islands of Sicily and Sardinia (Calabrò *et al*, 1998; Perna *et al*, 2000; Santarelli *et al*, 2001; Serraino *et al*, 2001).

Although sexual transmission is chiefly implicated in the high prevalence of KSHV among homosexual and bisexual men (Moore and Chang, 1998), other transmission routes have yet to be fully elucidated. KSHV is shed in saliva, like other viruses of the Herpes family (Wojcicki, 2003), and nonsexual transmission has been suggested to be the major route of transmission in Africa, where the infection is common in children (Mbulaiteye *et al*, 2003). Our findings concerning the importance of birth place, independently of place of residence, also suggest that in Italy early acquisition of KSHV infection is, or was, common in the middle-aged or elderly individuals with CKS in the present study. The excess of CKS in some Italian areas where wetlands and swamps are widespread (e.g. the Po delta, where Ferrara is located, and part of Sardinia) may have different explanations (Ascoli *et al*, 2001). These include a link between KS and malaria (Geddes *et al*, 1995) or, rather than malaria itself, the high-density of particular haematophagous insects (e.g. malaria vector *Anopheles*, black flies, sand flies, biting midges and mosquitoes). The practice of applying saliva to children's bite sites to relieve the itching and scratching may represent an efficient route of KSHV transmission (Coluzzi *et al*, 2003). Extensive land reclamation up to the 1960s and DDT-spraying in the early 1950s in malaria-endemic areas of Italy should have reduced this type of KSHV transmission (Coluzzi *et al*, 2003), but did not seem to have yet produced a decline of CKS, a disease chiefly of the elderly. Genetically increased susceptibility to KSHV infection and KS development in some Mediterranean populations, including those from Southern Italy, is also a possibility (Iscovich *et al*, 2000).

The significance of our findings depends on the accurate distinction of CKS from other neoplasms and KS variants. The majority (96%) of CKS reported here were histologically confirmed and, therefore, misclassification with sarcomas other than KS or with skin cancer can be ruled out. Misclassification of some AKS as CKS is of greater concern but should not account for our findings. AIDS registration in Italy is nationwide and has been shown to be almost complete (Ajdaicic-Gross *et al*, 2001). Moreover, in Italy, HIV-testing is routinely performed in patients with KS and CR staff reviewed medical records of all CKS cases. In very few KS (2%), not matched with RAIDS, HIV-positivity or history of illnesses typically related to HIV or AIDS were found. Few cases of KS would have arisen because of severe iatrogenic immune suppression but, in the three CRs that systematically collected details of organ transplantation, only 3% of KS unrelated to AIDS had occurred in organ recipients.

In conclusion, between 1985 and 1998 in Italy, CKS accounted for 97% of all KS in elderly people (⩾65 years) and a non-negligible fraction (42%) of KS in men between 39 and 64 years. Presently, there is no evidence that the improvements in the standard of living in Italy over the last decades are causing a decline in this disease. Separation of CKS from AKS will be important in studies of the disease, especially now that HIV-positive individuals may live into old age.

## Figures and Tables

**Table 1:** Observed (Obs) cases of classic and AIDS-related Kaposi's sarcoma (KS) by site of presentation in 15 Italian Cancer Registries, 1985–1998

	**Classic KS**		**AIDS-related KS**	
**ICD 10; Site of presentation**	**Obs**	**(%)^a^**	**Obs**	**(%)^a^**
C00–C14; lip, oral cavity and pharynx	9	(1.4)	29	(5.6)
C15–C26; digestive organs	9	(1.4)	20	(3.9)
C30–C39; respiratory and intrathoracic organs	5	(0.8)	2	(0.4)
*C44; skin*	551	(88.2)	444	(86.2)
C44.1–6; skin of face, scalp, neck, trunk, arm and shoulder	128	(23.2)	160	(36.0)
C44.7; skin of leg and hip	301	(54.6)	68	(15.3)
C44.8–9; skin, other or not specified	122	(22.2)	216	(48.6)
				
C49; connective, subcutaneous and other soft tissue	27	(4.3)	10	(1.9)
C51–C68; genital and urinary organs	15	(2.4)	5	(1.0)
Other and ill-defined sites	9	(1.4)	5	(1.0)
C80; unknown primary site	249		119	
				
All sites	874		634	

a Unknown primary site excluded from the calculation.

**Table 2:** Observed (Obs) cases, standardised incidence rates^a^ (IR), and 95% confidence intervals^b^ (CI) of classic Kaposi's sarcoma by gender in 15 Italian Cancer Registries, 1985–1998

	**Male**			**Female**		
**Cancer registry**	**Obs**	**IR**	**(95% CI)**	**Obs**	**IR**	**(95% CI)**
*North of Italy*						
Turin	86	1.22	(0.98–1.51)	35	0.43	(0.30–0.59)
Biella	2	0.44	(0.05–1.60)	5	0.90	(0.28–2.14)
Genoa	72	1.17	(0.91–1.48)	16	0.24	(0.14–0.39)
Varese	59	1.24	(0.94–1.60)	28	0.53	(0.35–0.76)
North East	28	0.66	(0.44–0.96)	16	0.32	(0.18–0.53)
Veneto	59	0.75	(0.57–0.97)	30	0.32	(0.21–0.45)
Parma	23	0.65	(0.41–0.97)	5	0.13	(0.04–0.30)
Modena	37	0.97	(0.68–1.33)	24	0.59	(0.38–0.88)
Ferrara	38	1.68	(1.18–2.31)	23	0.90	(0.57–1.36)
Romagna	38	0.70	(0.49–0.96)	26	0.44	(0.29–0.64)
						
*Centre of Italy*						
Macerata	6	0.45	(0.16–0.98)	3	0.23	(0.04–0.70)
Florence	54	0.63	(0.47–0.83)	29	0.30	(0.20–0.44)
Umbria	5	0.26	(0.08–0.61)	4	0.18	(0.05–0.46)
						
Total, North and Centre	507	0.88	(0.81–0.96)	244	0.38	(0.33–0.43)
						
*South of Italy*						
Sassari	60	4.69	(3.57–6.04)	20	1.65	(1.00–2.55)
Ragusa	28	1.47	(0.98–2.13)	15	0.92	(0.51–1.52)
						
Total, South	88	2.81	(2.25–3.46)	35	1.23	(0.85–1.71)
						
Total, Italy	595	0.98	(0.90–1.06)	279	0.41	(0.36–0.46)

a Age-standardised (per 100 000 population) according to Italian population, 1991.

b Computed according to Poisson distribution.

**Table 3:** Observed (Obs) cases of classic Kaposi's sarcoma, standardised incidence rates (IR)^a^, rate ratio and 95% confidence intervals^b^ (CI) by place of birth and place of residence among individuals of both genders in 12 Northern and Central Italian Cancer Registries^c^, 1985–1998

	**Place of birth**					
	**South and Islands^d^**		**North and Centre^e^**			
**Place of residence**	**Obs**	**IR**	**Obs**	**IR**	**Rate Ratio^f^**	**(95% CI)**
*North of Italy*						
Turin	85	2.08	34	0.37	5.6	(5.1–6.1)
Biella	0	0.00	7	0.78	0.0	–
Genoa	31	1.26	55	0.48	2.3	(2.0–2.5)
Varese	19	1.18	61	0.75	1.6	(1.4–1.8)
North East	12	2.66	29	0.38	7.0	(5.5–8.9)
Veneto	13	2.65	73	0.52	5.1	(4.3–6.2)
Parma	4	1.27	23	0.34	3.8	(2.0–7.0)
Modena	10	2.19	50	0.75	2.9	(2.2–3.9)
Ferrara	2	2.30	59	1.33	1.7	(0.6–4.9)
Romagna	6	1.82	56	0.53	3.4	(2.3–5.1)
						
*Centre of Italy*						
Florence	25	1.00	54	0.31	3.2	(2.8–3.6)
Umbria	3	1.30	5	0.14	9.5	(3.2–28.0)
						
Total, North and Centre	210	1.56	506	0.48	3.2	(3.2–3.3)

a Standardised by age and gender (per 100 000 population) according to Italian population, 1991.

b Computed according to Poisson distribution.

c Information is not available for Macerata Cancer Registry and is missing for 26 CKS cases.

d South and Islands includes Basilicata, Puglia, Campania, Calabria, Sicily and Sardinia.

e North and Centre includes Valle d'Aosta, Piedmont, Liguria, Lombardy, Trentino-Alto Adige, Veneto, Friuli-Venezia Giulia, Emila-Romagna, Tuscany, Lazio, Umbria, Marche, Abruzzo and Molise.

f South and Islands *vs* North and Centre.

## Appendix

*Cancer and AIDS Registry Linkage Study*: Pierluca Piselli (INMI ‘L Spallanzani’, Rome); Claudia Braga, Gary Clifford (IARC Lyon, France); Massimiliano Oggiano (Registro Tumori della Provincia di Sassari); Carmela Nicita (Registro Tumori Ragusa); Paolo Contiero (Registro Tumori Lombardia – Provincia di Varese); Stefano Rosso (Registro Tumori Piemonte, Torino); Maria Elisa Artioli (Registro Tumori della Provincia di Modena); Maria Antonietta Orengo (Registro Tumori Ligure, Genova); Pier Carlo Vercellino (Registro Tumori della Provincia di Biella); Loris Zanier (Registro Tumori del Friuli-Venezia Giulia); Fabio Vittadello (Registro Tumori dell'Alto Adige); Rosa Vattiato (Registro Tumori della Romagna, Forlì); Carmen Fiorella Stocco (Registro Tumori del Veneto, Padova); Eugenio Paci (Registro Tumori Toscano, Firenze); Lidia Serventi (Registro Tumori della Provincia di Parma); Franco Pannelli (Registro Tumori Provincia di Macerata, Camerino); Maria Gentilini (Registro Tumori della Provincia di Trento); Francesco La Rosa (Registro Tumori Umbro); Valerio Ramazzotti, Maurilio Natali (Registro Tumori di Popolazione della Provincia di Latina); Mario Fusco, Maurizio Montella (Registro Tumori della Provincia di Napoli); Stefano Boros (Istituto Superiore di Sanità, Roma)
